# Novel Caffeic Acid Phenethyl Ester-Mortalin Antibody Nanoparticles Offer Enhanced Selective Cytotoxicity to Cancer Cells

**DOI:** 10.3390/cancers12092370

**Published:** 2020-08-21

**Authors:** Jia Wang, Priyanshu Bhargava, Yue Yu, Anissa Nofita Sari, Huayue Zhang, Noriyuki Ishii, Kangmin Yan, Zhenya Zhang, Yoshiyuki Ishida, Keiji Terao, Sunil C. Kaul, Eijiro Miyako, Renu Wadhwa

**Affiliations:** 1 AIST-INDIA DAILAB, DBT-AIST International Center for Translational & Environmental Research (DAICENTER), National Institute of Advanced Industrial Science & Technology (AIST), Tsukuba, Ibaraki 305-8565, Japan; wang-jia0819@aist.go.jp (J.W.); bhargava.priyanshu89@gmail.com (P.B.); yu-yue@aist.go.jp (Y.Y.); sari-anissa@aist.go.jp (A.N.S.); zhang-huayue@aist.go.jp (H.Z.); n.ishii@aist.go.jp (N.I.); yan.kangmin@aist.go.jp (K.Y.); s-kaul@aist.go.jp (S.C.K.); 2 Graduate School of Life & Environmental Sciences, University of Tsukuba, Ibaraki 305-8575, Japan; zhang.zhenya.fu@u.tsukuba.ac.jp; 3 Biomedical Research Institute (BMRI), National Institute of Advanced Industrial Science & Technology (AIST), Ikeda 563-8577, Japan; 4 CycloChem Co., Ltd., 7-4-5 Minatojima-minamimachi, Chuo-ku, Kobe 650-0047, Japan; yoshiyuki.ishida@cyclochem.com (Y.I.); keiji.terao@cyclochem.com (K.T.); 5 KAUL-Tech Co. Ltd., 3-24 Nagakunidai, Tsuchiura City, Ibaraki 300-0810, Japan; 6 School of Materials Science, Japan Advanced Institute of Science & Technology, 1-1 Asahidai, Nomi, Ishikawa 923-1292, Japan; e-miyako@jaist.ac.jp

**Keywords:** CAPE, mortalin, internalizing antibody, nanoparticles, enhanced drug delivery, cancer therapy

## Abstract

Caffeic acid phenethyl ester (CAPE) is a key bioactive ingredient of honeybee propolis and is claimed to have anticancer activity. Since mortalin, a hsp70 chaperone, is enriched in a cancerous cell surface, we recruited a unique cell internalizing anti-mortalin antibody (MotAb) to generate mortalin-targeting CAPE nanoparticles (CAPE-MotAb). Biophysical and biomolecular analyses revealed enhanced anticancer activity of CAPE-MotAb both in in vitro and in vivo assays. We demonstrate that CAPE-MotAb cause a stronger dose-dependent growth arrest/apoptosis of cancer cells through the downregulation of Cyclin D1-CDK4, phospho-Rb, PARP-1, and anti-apoptotic protein Bcl2. Concomitantly, a significant increase in the expression of p53, p21^WAF1^, and caspase cleavage was obtained only in CAPE-MotAb treated cells. We also demonstrate that CAPE-MotAb caused a remarkably enhanced downregulation of proteins critically involved in cell migration. In vivo tumor growth assays for subcutaneous xenografts in nude mice also revealed a significantly enhanced suppression of tumor growth in the treated group suggesting that these novel CAPE-MotAb nanoparticles may serve as a potent anticancer nanomedicine.

## 1. Introduction

Propolis is a resinous mixture produced by honeybees by mixing their saliva with the botanical sources they live on. It has been used safely in traditional medicine for various health purposes over the past century and assigned a wide range of biological and pharmacological properties including anti-inflammatory, antibacterial, antiviral, antifungal, antiproliferative, and antioxidant activities [1,2,3,4,5,6]. More than 300 bioactive compounds have been identified in propolis [7]. Among these, phenolic acids, flavonoids, phenolic acid esters, terpenoids, steroids, and amino acids have been claimed to be the major bioactive components and explored in various pharmacological studies [8,9]. The chemical composition of propolis has been shown to vary with geographical localization and season [10]. New Zealand propolis has been shown to possess caffeic acid phenethyl ester (CAPE) as a predominant active component [11]. CAPE has been assigned as a potent anticancer component of propolis by in vitro studies using a large variety of cancer cell lines. Remarkably, it has been shown to be safe for normal cells [12,13,14,15,16,17,18,19]. Molecular studies have demonstrated that CAPE suppresses cancer cell proliferation via (i) inhibition of p70S6K and Akt signaling networks [20,21], (ii) activation of cell cycle arrest via Skp2, p53, p21^Cip1^, and p27^Kip1^ [22], (iii) inhibition of lipoxygenase [23], (iv) inhibition of nuclear factor kappa B (NF-κB) and activation of Fas [24,25], (v) apoptosis accompanied by activation of caspase-3/caspase-7 [18], (vi) involvement of the PI3-K/Akt and AMPK signaling pathways [26], and (vii) suppression of β-catenin associated signaling pathway and p53-dependent apoptosis mediated by p38 MAPK [27,28]. Consistent with the in vitro reports, the anticancer activity of CAPE was also established by in vivo studies in mice [26,29]. Although these reports have endorsed the presence of bioactivities in CAPE and suggested it as a natural drug for cancer prevention and therapy, its use has been limited due to instability and degradation by cellular esterases [30,31]. Hence, further investigations are warranted to design its formulations/derivatives that overcome the issues of its bioavailability and degradation. We earlier showed that the CAPE when complexed with γCD is protected against the degradation by esterases and possesses high stability and anticancer activity [30,31].

Among the various challenging issues of the pharmaceutical formulation of CAPE, the most important is to improve the poor water solubility and bioavailability at the target site for the therapeutic effect of this hydrophobic compound. Of the numerous strategies including recruitment of carriers such as antibodies, liposomes, and nanoparticles while drug designing, nanomedicine is one of the most promising medical applications of nanotechnology for diagnostic and therapeutic purposes. It can help to improve the efficacy, safety, specificity, tolerability, and therapeutic index of corresponding drugs [32,33,34,35,36]. The nanocarriers can effectively encapsulate and release various small molecules by the modification of physicochemical properties (size, shape, composition, surface charge, ligands, hydrophilicity, and hydrophobicity) [37,38,39]. Recently, polymers have gained much attention in the field of drug delivery. For example, polyethylene glycol (PEG)-coated polymer nanocarriers have shown to boost the therapeutic action of drugs by passive targeting based on enhanced permeability and retention (EPR) effect [40,41]. EPR effect has been reported to play an important role in the accumulation of nanocarriers in tumor cells [42]. The engineered molecules and their derivatives have been able to enhance the therapeutic potential through efficiently targeting drug delivery. However, it requires the corresponding optimization in sufficient quantities for the targeted and uniform delivery of the nanocarriers to tumors. Therefore, an ideal nanocarrier must be very selective for a receptor that is highly or differentially expressed on the surface of tumor cells for the targeted release of the carried drug.

Mortalin, a highly conserved heat shock chaperone, is located in mitochondria, endoplasmic reticulum, plasma membrane, cytoplasmic vesicles, and cytosol with different subcellular distribution patterns in normal and transformed human cells [43,44,45]. Mortalin has been extensively reported to be enriched in cancer cells and contributes to carcinogenesis [46,47,48]. Aggressive and metastatic cancer cells have also been shown to express mortalin on their cell surface that can bind to unique anti-mortalin antibodies (MotAb) and cause their internalization into the cells [49,50,51]. Due to their unique cell internalization property, MotAb was proposed as a promising vehicle for targeted delivery of candidate anticancer drugs [51]. In this context, we predicted that in comparison to naked CAPE, CAPE-MotAb nanoparticles may offer enhanced delivery, selectivity, and cytotoxicity to cancer cells. In the present study, we have generated CAPE-MotAb nanoparticles and demonstrate their physical and functional characterization that indeed prove their higher anticancer efficacy in vitro and in vivo.

## 2. Materials and Methods

### 2.1. Preparation of CAPE-MotAb

Caffeic acid phenethyl ester (CAPE; 10 mg) (SynphaTec Japan Co., Ltd, Osaka, Japan) and DSPE-PEG-NHS (3-(N-succinimidyloxyglutaryl) aminopropyl, polyethyleneglycol-carbamyl distearoylphosphatidyl-ethanolamine; MW 2000; 50 mg) (SUNBRIGHT DSPE-020GS; Yuka Sangyo, Tokyo, Japan) were dissolved in 10 mL PBS (phosphate-buffered saline) buffer and stirred at room temperature for about 1 h. The mixture was then sonicated in ice-cold water for 10 min using a pulse-type sonicator (VCX-600; Sonics, Danbury, CT, USA). A total of 400 μg (2.6 nM) of cell internalizing anti-mortalin monoclonal antibody (MotAb; Clone C1-3) raised in our laboratory was immediately added into this reaction and stirred at 4 °C overnight. In order to avoid the existence of free antibody, the ratio of antibody (2.6 nM) to DSPE-PEG-NHS (25 μM) in the mixture was kept at 1:9600 ratio. The mixture was centrifuged at 1000 rpm for 30 min. The supernatant was used for subsequent experiments. The CAPE-PEG nanoparticles (without adding MotAb) were prepared by the same protocol and used as a negative control.

### 2.2. Characterization of CAPE-MotAb 

The MotAb loaded in the nanocapsules was detected by non-reducing SDS-PAGE analysis. Briefly, samples were prepared using 1 X SDS sample buffer without adding β-mercaptoethanol and loaded into the wells of the gel, along with a molecular weight marker. The gel was stained with 0.1% Coomassie brilliant blue G-250. The spectral profiles and concentrations of CAPE in nanocapsules were measured at room temperature on an ultraviolet-visible-near infrared (UV-Vis-NIR) spectrophotometer (UV-2600; Shimazu, Tokyo, Japan). The absorption spectra were measured from 200 to 800 nM. The structure and morphology of CAPE-MotAb nanoparticles were visualized by high-resolution transmission electron microscope (Tecnai F20 TEM; FEI Company, Eindhoven, Netherlands). An aliquot of 10-fold diluted CAPE-MotAb solution was applied onto a TEM specimen grid covered with a thin carbon support film of which the surface was made hydrophilic by a hydrophilic treatment device, HDT-400 (JEOL DATUM, Tokyo, Japan). The excess solution was blotted by a filter paper followed by negatively staining with 2% uranyl acetate. Samples were carefully observed on a TEM operated at an accelerating voltage of 120 kV. TEM images were recorded by making use of the slow-scan CCD camera (Retractable Multiscan Camera; Gatan, Inc., Pleasanton, CA, USA) at magnifications 25 k, and 62 k. The size distribution was analyzed using Image-J from TEM images software (National Institute of Health, Bethsda, MD, USA) (7500 particles were measured).

### 2.3. Encapsulation and Loading Efficiency of CAPE-MotAb 

CAPE-MotAb was prepared with different weight ratios of purified CAPE powder and DSPE-PEG-NHS polymers as indicated. After using the nanocapsules preparation procedure, as mentioned above, the different CAPE-MotAb were left undisturbed for more than 24 h. The supernatant in the mixture was collected and the amount of CAPE was measured by UV-Vis-NIR spectrophotometer. This amount of CAPE was determined against a reference of its calibration curve in the region of 335 nM. The encapsulation and loading efficiency of CAPE into CAPE-MotAb was estimated as given below:

$$\mathrm{Encapsulation}\;\mathrm{efficiency}\;\left(\mathrm{EE}\%\right)=\frac{{\mathrm{CAPE}}_{\mathrm{supernatant}}\;\left(\mathrm{mg}\right)}{\mathrm{Total}\;\mathrm{adding}\;\mathrm{CAPE}\;\mathrm{amount}\;\left(\mathrm{mg}\right)}\times 100\%$$

$$\mathrm{Loading}\;\mathrm{efficiency}\;\left(\mathrm{LE}\%\right)=\frac{{\mathrm{CAPE}}_{\mathrm{supernatant}}\;\left(\mathrm{mg}\right)}{\begin{array}{c} \mathrm{Total}\;\mathrm{adding}\;\mathrm{CAPE}\;\mathrm{and}\;\mathrm{DSPE}-\mathrm{PEG}-\mathrm{NHS}\\ \mathrm{amount}\;\left(\mathrm{mg}\right) \end{array}}\times 100\%$$

### 2.4. Cell Lines and Culture

Human cancer and normal cells purchased from the National Institute of Physical and Chemical Research (RIKEN, Japan) and the Japanese Collection of Research Bioresources (JCBR), Japan were used in the present study. Human breast carcinoma (MCF7, MDA-MB-231, MDA-MB-453, and T47D), osteosarcoma (U2OS and Saos-2), colon carcinoma (COLO-320, DLD-1, HCT116, and HT-29), lung carcinoma (A549 and H1299), cervical carcinoma (HeLa, ME-180, CaSki, and SKG-II), liver carcinoma (Huh6 and Huh7), fibrosarcoma (HT1080), ovarian carcinoma (SKOV-3), and normal human lung fibroblast (MRC5) were cultured in Dulbecco’s Modified Eagle’s Medium (DMEM; Gibco BRL, Grand Island, NY, USA) supplemented with 10% (v/v) fetal bovine serum (Gibco BRL) and 1% (v/v) penicillin/streptomycin at 37 °C in a humidified incubator with 5% CO_2_ and 95% air.

### 2.5. Cell Viability and Morphological Observations 

The short-term cell cytotoxicity was evaluated by MTT 3-(4, 5- dimethylthiazol-2-yl)-2, 5-diphenyltetrazolium bromide assay as described earlier [51]. Cells (5 × 10^3^ cells/well) were seeded in 96-well plates and allowed to adhere overnight with incubation at 37 °C in a CO_2_ incubator. The cells were then treated with different concentrations of CAPE, CAPE-PEG, CAPE-MotAb, DSPE-PEG-NHS, and MotAb as indicated, followed by addition of 10 μL of MTT (5 mg/mL; Sigma-Aldrich, St. Louis, MO, USA) to each well and further incubated at 37 °C for 4 h. The medium with MTT was replaced with 100 μL of dimethyl sulfoxide (DMSO). The absorbance of the plates was then measured on a microplate reader (Infinite M200 PRO; TECAN, Männedorf, Switzerland) at 570 nM. In order to observe the morphology, cells (2 × 10^5^ cells/well) were seeded in 6-well plates and treated with CAPE-PEG, CAPE-MotAb, DSPE-PEG-NHS and MotAb followed by observation under the phase-contrast microscope (Nikon Eclipse TE300; Nikon, Tokyo, Japan).

### 2.6. Colony Formation Assay 

The long-term cytotoxicity effect of CAPE-PEG, CAPE-MotAb, DSPE-PEG-NHS, and MotAb on human cells was evaluated by colony-forming assay. Cells (500 per well) were seeded in 6-well plates and allowed to adhere to the substratum for overnight followed by 12-h treatment with CAPE-PEG, CAPE-MotAb, DSPE-PEG-NHS, and MotAb. The cells were cultured in normal medium with a regular change of medium every third day until colonies were formed. Colonies were washed thrice with cold PBS and fixed with methanol/acetone (1:1, *v*/*v*) at 4 °C for 10 min. Fixed colonies were again washed thrice with cold PBS, stained with 0.1% crystal violet solution (Wako, Osaka, Japan) overnight, de-stained with water, and left open for air drying. The plates were then subjected to photography, scanning by EPSON scanner, and colony counting.

### 2.7. Flow Cytometry Analysis

Mortalin expression was detected on the surface of various cancer cell lines using the Cell Surface Staining Flow Assay Kit (Novus Biologicals, LLC, Englewood, CO, USA) following the manufacturer’s instructions. The expression analysis was performed using Guava PCA flow cytometer (Millipore, Billerica, MA, USA) following the manufacturer’s protocol. 

For cell cycle analysis, cells (2 × 10^5^ cells/well) treated with CAPE-PEG and CAPE-MotAb were harvested using trypsin after 24-h incubation. Cell pellets were washed with cold PBS and fixed with 70% cold ethanol at −20 °C overnight. The fixed cells were centrifuged (500× *g*) for 5 min at 4 °C and washed twice with cold PBS followed by resuspension in 1 mL cold PBS. RNase A was used to remove RNA and to avoid false DNA-PI staining. RNAse was added to the final concentration of 100 μg/mL and incubated at 37 °C for 1 h followed by centrifugation (500× *g*) at 4 °C for 5 min. The supernatant was replaced with 200 μL Guava Cell Cycle reagent (Millipore), incubated in the dark at room temperature for 30 min, and then subjected to the Guava PCA flow cytometer (Millipore). 

The data obtained from these two experiments were further analyzed using FlowJo software (version 7.6; LLC, USA).

### 2.8. Apoptosis Assay

Cell apoptosis was determined by Annexin-V and 7-aminoactinomycin (7-AAD) double staining. Cells were seeded at a density of 2 × 10^5^/well followed by treatments as indicated. Suspended and attached cells were harvested with trypsin and centrifuged (1200 rpm) at 4 °C for 2 min. Cells were resuspended in medium to make the concentration equal to 1 × 10^6^ cells/mL. One hundred microliters of each cell suspension were incubated with 100 μL of Guava Nexin Reagent (Millipore) at room temperature in the dark for 20 min and subjected to the Guava PCA flow cytometer (Millipore). Apoptotic cells were determined with FlowJo software (version 7.6; LLC, USA).

### 2.9. Western Blotting 

Cellular protein from the plasma membrane fraction was extracted using the plasma membrane protein extraction kit (ab65400; Abcam, Cambridge, MA, USA). The protein was further incubated with anti-mortalin antibody for Western blotting. Cells were lysed in radioimmunoprecipitation assay buffer (RIPA buffer; Thermo Fisher Scientific, Waltham, MA, USA) supplemented with a protease inhibitor cocktail (Roche Applied Science, Mannheim, Germany). Protein concentrations were determined using the Pierce BCA Protein Assay kit (Thermo Fisher Scientific). Western blotting was performed as described earlier [52] with the following primary antibodies: Anti-p53 (FL-393), anti-cyclin D1 (CD1.1), anti-CDK4 (C-22), anti-PARP-1 (H-250), anti-Caspase 9 (H-83), anti-Bax (N-20), anti-MMP2 (H-76), anti-MMP3/10 (FL-10), anti-Vimentin (V9) from Santa Cruz Biotechnology (Santa Cruz, CA, USA), anti-p21^WAF-1^ (12D1), anti-pRb (ser780), anti-Bcl-2 (2876S), anti-MMP9 (G657), anti-hnRNP-k (R332) from Cell Signaling Technologies (Danvers, MA, USA), anti-Caspase 3 (610322; BD Transduction Laboratories, San Diego, CA, USA), anti-Cytochrome C (ab133504; Abcam, Cambridge, MA, USA), and anti-CARF (FL-10) [53]. Anti-β-actin (AC-15; Abcam, Cambridge, MA, USA) was used as an internal loading control. Quantitation results of the protein expression were determined using ImageJ software (National Institute of Health).

Tumor tissues were obtained from sacrificed mice as mentioned in Section 2.14. Tumors were washed with PBS thrice and lysed in RIPA buffer containing protease inhibitor cocktail by homogenization using a glass tissue homogenizer. The tumor lysates were collected by centrifugation at 13,000 rpm for 30 min at 4 °C and then subjected to Western blotting as mentioned above. 

### 2.10. Fluorescence Microscopy 

Cells (1 × 10^5^ cells/well) were seeded on 18-mm glass coverslips placed in 12-well culture dishes. After incubation with CAPE-PEG, CAPE-MotAb, DSPE-PEG-NHS, and MotAb, cells were washed thrice with cold PBS, fixed with methanol/acetone (1/1, v/v) at 4 °C for 10 min and then again washed thrice with cold PBS. Coverslips containing cells were then incubated with Alexa Fluor-conjugated secondary antibodies (Thermo Fisher Scientific). Nuclei were stained with Hoechst 33342 (1 µg/mL; Thermo Fisher Scientific) for 10 min. After three washes with PBS, the cells were mounted and visualized under a Carl Zeiss microscope (Axiovert 200 M; Tokyo, Japan). Quantitation of the images was obtained by ImageJ software (National Institute of Health).

### 2.11. Cellular Uptake 

Cellular uptake was quantified as described elsewhere [54]. The calibration curve of CAPE amount was obtained based on the absorbance at 335 nM using various concentrations of CAPE-PEG. Cells (2 × 10^5^ cells/well) were seeded in 6-well plates and incubated overnight. After adherence, cells were treated with CAPE-PEG or CAPE-MotAb in the medium for 12 h. Cell pellets were harvested and lysed in RIPA buffer. After three washes with PBS, cell pellets were collected and resuspended in RIPA buffer followed by 30 min of sonication. One portion of the cell lysate was used for protein estimation (Pierce BCA Protein Assay kit; Thermo Fisher Scientific). The remaining cell lysate was used to measure the absorbance at 335 nM in triplicate using a microplate reader (TACAN). The concentration of CAPE in cell lysates (CAPE/protein) was estimated based on the corresponding calibration curves.

### 2.12. Wound Scratch Assay

The wound scratch assay was used to examine the cell motility. Cell monolayers were wounded by uniformly scratching the surface with a 200-μL tip. Movement of the control and treated cells in the scratched area was then periodically monitored under a phase-contrast microscope with a 10 X phase objective (Nikon Eclipse TE300; Nikon, Tokyo, Japan). The migration capacity of the cells was calculated by measuring the percentage of open area in 6 to 10 randomly captured images.

### 2.13. Cell Invasion Assay

Cell invasion assays were carried out using Corning BioCoat™ Matrigel Invasion Chamber (24-well plate 8.0 Micron; BD Biosciences, Tokyo, Japan) following the manufacturer’s instructions. For measurement, cells were stained, and the invading cells were counted by photographing the membrane in a phase-contrast microscope (Nikon Eclipse TE300; Nikon, Tokyo, Japan).

### 2.14. In Vivo Xenograft Assay

Four- to five-week-old female BALB/c AJcl-nu/nu nude mice were injected subcutaneously in both flanks with 5 × 10^6^ human lung carcinoma A549 cells in 100 μL culture medium. The study was carried out in three groups with 4 mice each. The first group served as a control. The second group was treated with CAPE, while the third group was treated with CAPE-MotAb. An equivalent dosage of CAPE (200 mg/kg) in its free form (in order to compare with the already reported anti-cancer activity [30] and in nanoparticles) was given until cells formed tumors for about 10 days. PBS was used as a negative control. Intraperitoneal injection was given every alternate day. Mice body weight and tumor size was monitored every 2 days. Subcutaneous tumor volume was calculated according to the formula: V = L × W^2^/2, where L was the length and W was the width of the tumor, respectively. At the end of the experiment, mice were sacrificed, the tumors were taken out and weighed. The tumor growth inhibition rate was calculated as % using (1 − Wt/Wc) × 100% (Wt was the mean volume of treated tumors and Wc was the mean volume of control tumors). This study was carried out in strict accordance with the recommendations from the Animal Experiment Committee, Safety and Environment Management Division, National Institute of Advanced Industrial Science and Technology (AIST), Japan (Approval Number 2019-0025).

### 2.15. Statistical Analysis

All the experiments were performed at least three independent times and results were presented as mean ± standard deviation (SD). The degree of statistical significance among the control and sample groups were calculated by an unpaired t-test (GraphPad Prism GraphPad Software, San Diego, CA, USA). Significant values have been represented as * *p* < 0.05, ** *p* < 0.01, and *** *p* < 0.001.

## 3. Results

### 3.1. Generation and Characterization of CAPE-MotAb Nanoparticles

**Figure 1:**
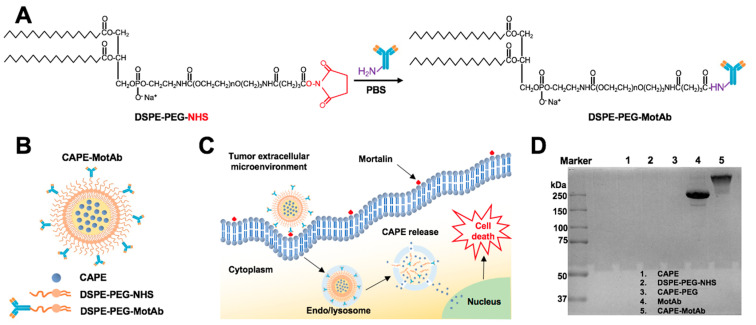

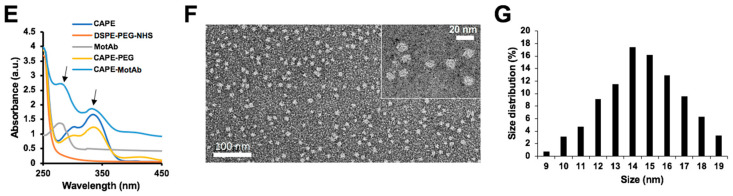
Schematic illustration of the construction and characteristics of CAPE-MotAb nanoparticles for targeted drug delivery. (**A**) MotAb modified with DSPE-PEG-NHS. (**B**) Structure of mortalin-targeted CAPE-MotAb nanoparticles formed by self-assembly of amphiphilic block copolymers (DSPE-PEG-NHS) with MotAb. (**C**) General mechanism of targeted action by CAPE-MotAb for cancer treatment: the nanocapsules with long blood circulation times get accumulated at the tumor region through passive targeting achieved by EPR effect and subsequently internalized by tumor cells via mortalin-mediated endocytosis. The low pH in endo/lysosomes offers an optimal environment to facilitate the CAPE escape to the cytoplasm by decomposing micelles, thus resulting in cell death. (**D**) Non-reducing SDS-PAGE analysis of CAPE, DSPE-PEG-NHS, CAPE-PEG, MotAb, and CAPE-MotAb. MotAb appeared at MW ~250-kDa, CAPE-MotAb was seen at higher molecular weight suggesting the successful conjugation of MotAb to DSPE-PEG-NHS. (**E**) UV-Vis-NIR absorption spectra of CAPE-MotAb. Characteristic peaks of MotAb at 280 nM and CAPE at 335 nM (marked with black arrows) were simultaneously observed in CAPE-MotAb showing successful encapsulation of CAPE in polymeric micelles. (**F**) Representative TEM image of CAPE-MotAb (a magnified inset is shown in the top right corner). (**G**) Quantitative size distribution of CAPE-MotAb shown as monodisperse and spherical in shape with a diameter ranging from 9 to 19 nM.

Polymeric micelles have attracted considerable attention as an effective delivery system for anticancer drugs that face poor water solubility issues [55,56]. Polyethylene glycol (PEG) is the most commonly used hydrophilic segment of polymeric micelles due to its biocompatibility and biodegradability [57]. Herein, we employed phospholipid PEG conjugates that can react with primary amine groups (DSPE-PEG-NHS) and anti-mortalin antibody (MotAb) to encapsulate CAPE in PEG-stabilized polymeric micelles and explored their characteristics (Figure 1A). The schematic illustration of CAPE-MotAb structure is shown in Figure 1B. The polymeric micelles containing CAPE were easily synthesized through a unique self-assembly behavior of amphiphilic block copolymers that have polar or hydrophilic groups as well as nonpolar or hydrophobic portions when dissolved in the solvent. In a hydrophilic solvent, the hydrophobic portions are clustered in a core, away from the solvent and the hydrophilic portions are aligned towards the solvent [58]. Hydrophobic CAPE was encapsulated in the nanoparticles composed of an inner hydrophobic domain (DSPE) and an outer hydrophilic part (PEG-modified with NHS). CAPE-MotAb was expected to have a prolonged circulation time, actively enter and accumulate at the tumor site, and have high loading capacity. Once in the tumor, these CAPE-MotAb nanoparticles were anticipated to rapidly release CAPE in acidic endo/lysosomes and subsequently deliver the drug to the cytoplasm and nucleus (illustrated in Figure 1C). We subjected the nanoparticles to non-reducing SDS-PAGE analysis (Figure 1D). As shown, the antibody was visible at the ~250-kDa molecular weight. Of note, the CAPE-MotAb nanoparticles showed higher molecular weight suggesting successful conjugation of MotAb to DSPE-PEG-NHS. The UV-Vis-NIR spectrum of CAPE-MotAb showed characteristic peaks of MotAb at 280 nM and CAPE at 335 nM confirmed the successful encapsulation of CAPE in MotAb-conjugated polymeric micelles (Figure 1E). The encapsulation efficiency of CAPE improved with an increasing amount of DSPE-PEG-NHS and reached the highest value of 84.88% ± 8.66% at 1:20 ratio of CAPE to DSPE-PEG-NHS (Table 1). The loading efficiency of CAPE reached the highest value of 19.65% ± 0.96% when CAPE and DSPE-PEG-NHS were used in a 1:1 ratio and found to decrease with an increase in polymer amounts (Table 2). The encapsulation and loading efficiency were both satisfactory with a ratio of 1:5 for CAPE and DSPE-PEG-NHS; hence it was selected as the optimum ratio for further experiments. These results strongly suggested that the DSPE-PEG-NHS could efficiently solubilize CAPE in water. As size and morphology have a wide influence on the biological applications of nanoparticles, we examined these aspects by transmission electron microscopy (TEM). The TEM observations revealed that CAPE-MotAb are monodisperse with spherical morphology (Figure 1F). We also calculated the size distribution of these nanoparticles from the TEM images and found that after conjugation with DSPE-PEG-NHS and MotAb, the nanoparticles are in the size ranging from 9 to 19 nm (Figure 1G). Furthermore, we examined the stability of CAPE-MotAb nanoparticles by UV-Vis-NIR spectrum of CAPE and Mot Ab at 335 nm and 280 nm, respectively. As shown in Figure S1, CAPE-MotAb nanoparticles were found to be stable even after eight days of incubation at 4 °C. Having confirmed the easy preparation, high stability, and reproducibility of CAPE-MotAb by multiple experiments, we then evaluated the in vitro and in vivo targeting efficiency, cytotoxicity, and anticancer properties of CAPE-MotAb nanoparticles.

### 3.2. CAPE-MotAb Showed Enhanced Selective Cytotoxicity in Cancer Cells 

**Figure 2:**
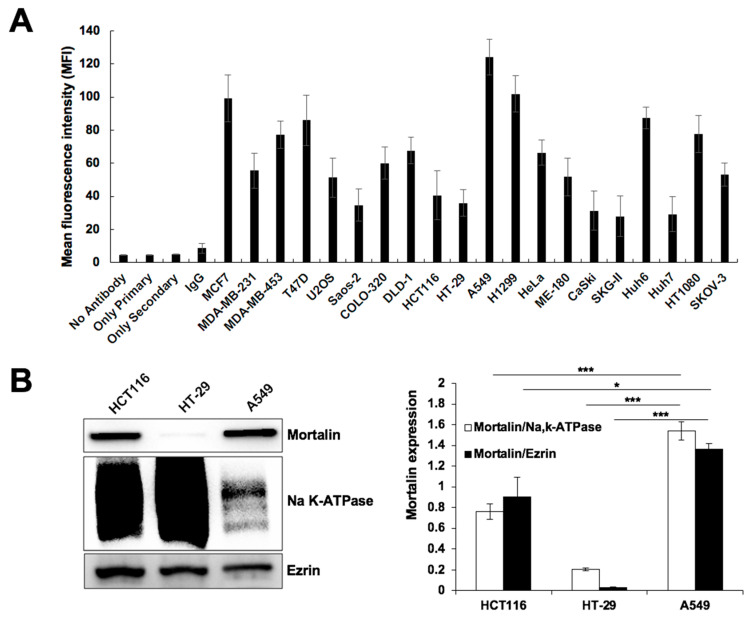
Cell surface expression of mortalin in different cancer cell lines. (**A**) Mean fluorescence intensity (MFI) of mortalin. FACS analysis showed cell surface expression, although to a variable extent, of mortalin in various cancer cells. Values are expressed as a median. (**B**) Western blotting for mortalin in the plasma membrane fractions of HCT116, HT-29, and A549 cells is shown; NaK-ATPase and Ezrin were used as the loading control and irrelevant membrane protein control, respectively. Quantitation data of Western blotting from three independent experiments is shown on the right (mean ± SD, *n* = 3), * *p* < 0.05, *** *p* < 0.001 (Student’s t-test).

In order to test the targeting efficacy of CAPE-MotAb, we first examined the expression level of mortalin on the cell surface of various cancer cells by fluorescence-activated cell sorting (FACS) using a specific anti-mortalin antibody. Quantitative estimation of the mortalin expression is summarized in Figure 2A. Among the cell lines analyzed, whereas MCF7, A549, and H1299 showed a higher level of mortalin expression at the cell surface; Saos-2, HCT116, HT-29, CaSki, SKG-II, and Huh7 showed a lower level. We also confirmed the level of mortalin expression on the cell surface by performing Western blotting on the plasma membrane fractions of HCT116, HT-29, and A549 cells. As shown in Figure 2B, among these three cell lines, A549 (lung carcinoma) showed a considerably higher level of cell surface expression of mortalin than HCT116 and HT-29 cells. Based on these expression data, we selected A549, HCT116, and HT-29 cells for further experiments.

**Figure 3:**
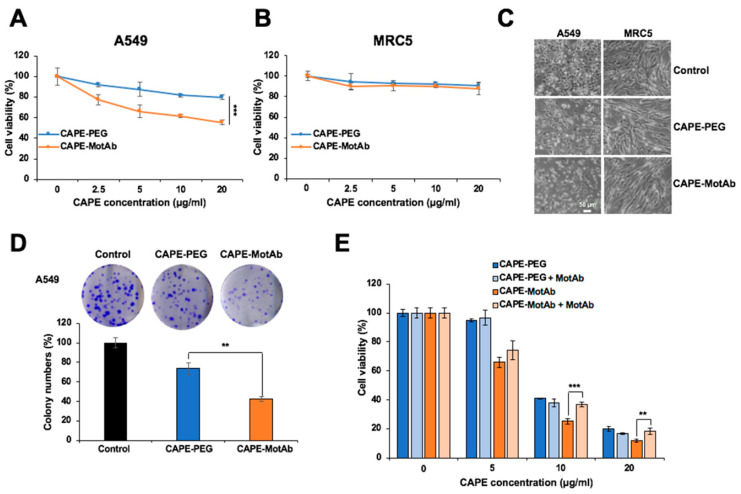
Enhanced selective cytotoxicity of CAPE-MotAb in vitro. (**A**) Cell viability assay indicated dose-dependent cytotoxicity of CAPE-PEG and CAPE-MotAb in A549 cancer cells treated for 24 h. CAPE-MotAb enhanced cancer-killing activity of CAPE in A549 cells, but not in MRC5 normal cells (**B**) (mean ± SD, *n* = 3), *** *p* < 0.001 (Student’s t-test). (**C**) Phase-contrast images showing toxicity of CAPE-MotAb (CAPE concentration: 20 μg/mL) in human cancer cells; MRC5 normal cells did not show significant toxicity after 24 h incubation. (**D**) A stronger reduction in colony-forming ability was observed in cells treated with CAPE-MotAb (CAPE concentration: 20 μg/mL). Quantitation from three independent experiments (mean ± SD, *n* = 3), ** *p* < 0.01 (Student’s t-test). (**E**) Cell viability of A549 cells treated with CAPE-PEG and CAPE-MotAb with/without MotAb (0.8 μg/mL) for 48 h (mean ± SD, *n* = 3), * *p* < 0.05, ** *p* < 0.01, *** *p* < 0.001 (Student’s t-test). CAPE-MotAb caused a dose-dependent decrease in cell viability, partially compromised by preincubation/simultaneous with MotAb.

We next examined the cytotoxicity of CAPE-PEG and CAPE-MotAb in A549 and MRC5 cells by MTT assay. An equal amount of MotAb was used as a negative control. Incubation of cells with MotAb did not cause any toxicity to either A549 or MRC5 cells (Figure S2). CAPE-MotAb treated cells showed dose- and time-dependent inhibition of proliferation as compared to free CAPE and CAPE-PEG (Figure 3A and Figure S3). Of note, A549 cells showed significantly higher toxicity to CAPE-MotAb as compared to CAPE and CAPE-PEG both at 24-h and 48-h treatments. On the other hand, HCT116 and HT-29 cells that possessed a lower level of cell surface expression of mortalin did not show significantly enhanced toxicity to CAPE-MotAb as compared to CAPE/CAPE-PEG (Figure S3). Furthermore, there was no difference in viability in MRC5 cells subjected to equivalent doses of CAPE-PEG or CAPE-MotAb (Figure 3B). Cell morphology as observed under the microscope also supported the toxicity of CAPE-MotAb in A549 cancer cells and negligible toxicity in MRC5 normal cells (Figure 3C). We next performed a colony formation assay to investigate the long-term effect of CAPE-PEG and CAPE-MotAb on cell proliferation. A549 cells when treated with CAPE-MotAb for 12 h showed a greater reduction in colony-forming efficiency (Figure 3D) as compared with CAPE-PEG treated cells. Of note, MotAb when added along with CAPE-PEG, without making a complex, failed to cause an increase in cytotoxicity at all doses of CAPE examined (Figure 3E). Furthermore, and as expected, the pre-incubation of CAPE-MotAb nanoparticles with MotAb compromised its cytotoxicity (Figure 3E).

### 3.3. CAPE-MotAb Showed Selective Uptake in Cancer Cells 

**Figure 4:**
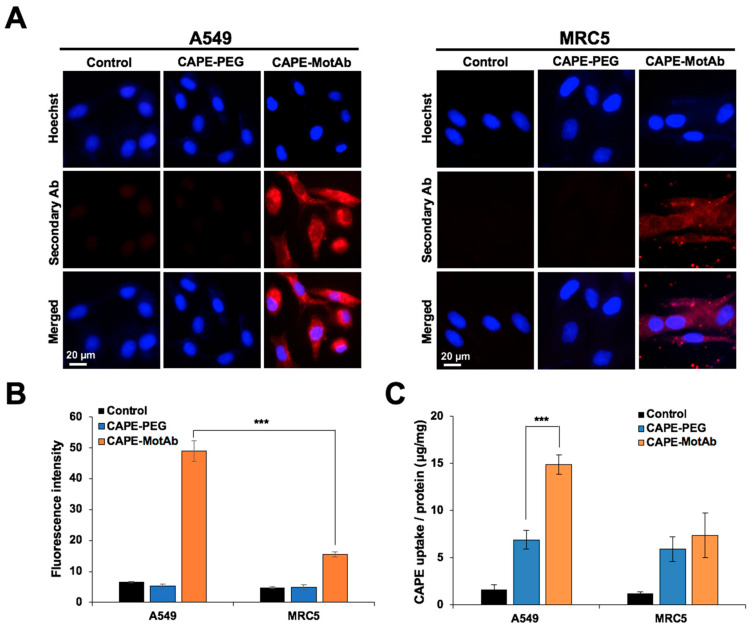
Selective cellular uptake of CAPE-MotAb in cancer cells by targeting mortalin. (**A**) Fluorescence microscopy images of A549 and MRC5 cells treated with CAPE-PEG and CAPE-MotAb followed by staining with Alexa Fluor^TM^ 594-tagged secondary antibody. The nuclei were stained with Hoechst. Higher cellular uptake efficiency of CAPE-MotAb was observed in A549 cells. (**B**) Quantitation of mortalin expression from fluorescence images (mean ± SD, *n* = 3), *** *p* < 0.001 (Student’s *t*-test). (**C**) Quantitative analysis of cellular uptake of CAPE-PEG and CAPE-MotAb in A549 and MRC5 cells (mean ± SD, *n* = 3), *** *p* < 0.001 (Student’s *t*-test). CAPE-MotAb treated A549 cells exhibited higher CAPE accumulation as compared to MRC5 cells treated with the same nanoparticles. A549 and MRC5 cells for all above experiments were treated with an equivalent dose of CAPE (20 µg/mL) for 12 h.

We next examined the uptake of CAPE-MotAb nanoparticles in A549 and MRC5 cells by fluorescence microscopy. Cells were incubated with CAPE-PEG, CAPE-MotAb, DSPE-PEG-NHS, and MotAb in culture medium at 37 °C for 12 h followed by staining with secondary antibody; the nucleus was stained with Hoechst (blue). As shown in Figure 4A and Figure S4, preferential internalization of MotAb in A549 cells was confirmed [51]; MRC5 cells showed only negligible internalization. In line with this data, the internalization of CAPE-MotAb nanoparticles in A549 cells was confirmed with secondary antibody staining (Figure 4A); MRC5 cells showed negligible staining (Figure 4B). Furthermore, A549 cells showed a time-dependent (12 to 48 h) increase in internalization as determined by MotAb fluorescence endorsing time-dependent cellular uptake of CAPE-MotAb nanoparticles (Figure S5).

In addition to the fluorescence microscopy, the uptake amount of CAPE in A549 and MRC5 cells was also determined to calculate the relative uptake efficiency and evaluate the cellular uptake of CAPE-PEG and CAPE-PEG-MotAb. The lysates from control and treated cells were subjected to spectrophotometric measurement of CAPE and the total protein. Here, CAPE uptake efficiency was defined as the amount of CAPE versus the total amount of protein. The uptake efficiency was found to be 6.9 μg/mg for CAPE-PEG and 14.9 μg/mg for CAPE-MotAb for A549 cells for 12-h treatment showing a 2.2-fold increase in uptake (Figure 4C). Remarkably, such an increase was not detected in MRC5 cells (Figure 4C). These results were consistent with the fluorescence imaging results and that further confirmed the cancer cell-specific targeting of CAPE-MotAb nanoparticles.

### 3.4. CAPE-MotAb Caused Stronger Cell Cycle Arrest and Apoptosis in Cancer Cells

**Figure 5:**
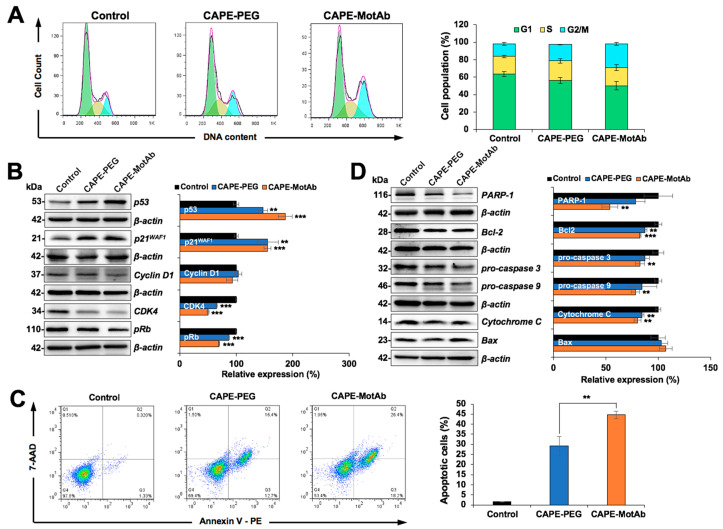
Stronger cell cycle arrest and apoptosis induced by CAPE-MotAb in A549 cells. (**A**) Cell cycle analysis of CAPE-MotAb treated A549 cells after 24-h incubation showing an increase in cell population in the G2/M phase in comparison to CAPE-PEG treated A549 cells. Quantitation of cell population from three independent experiments. (**B**) Western blot for cell cycle regulatory proteins (p53, p21^WAF1^, Cyclin D1, CDK4, and pRb) after 48-h incubation of CAPE-MotAb. Quantitation of the results is shown on the right (mean ± SD, *n* = 3), ** *p* < 0.01, *** *p* < 0.001 (Student’s t-test to control). (**C**) Apoptosis analysis from flow cytometry after 48-h incubation showing a sharp increase in apoptotic cells when treated with CAPE-MotAb. Quantitation of apoptotic cells from three independent experiments (mean ± SD, *n* = 3), ** *p* < 0.01 (Student’s t-test). (**D**) Western blot for apoptotic proteins (PARP-1, Bcl-2, pro-caspase 3, pro-caspase 9, Cytochrome C, and Bax) after 48-h incubation of cells with CAPE-MotAb. Quantitation of the results is shown on the right (mean ± SD, *n* = 3), ** *p* < 0.01, *** *p* < 0.001 (Student’s t-test to control). A549 cells were treated with an equivalent dose of CAPE (10 µg/mL) for all the above experiments.

In order to determine the biological activity of CAPE-MotAb nanoparticles, we performed cell cycle analysis. Control and treated A549 cells were subjected to flow cytometry analysis. We found that when A549 cells were treated with CAPE-MotAb there was a significant increase in cell population at G2/M phase (Figure 5A). The proportion of G2/M phase increased from 14% in control to 19% and 27% in CAPE-PEG and CAPE-MotAb treated cells, respectively, suggesting the enhancement of CAPE potency to cause growth arrest when conjugated with MotAb (Figure 5A). To further investigate the molecular mechanism of CAPE-MotAb-induced growth arrest, we analyzed the expression of various proteins (p53, p21^WAF1^, cyclin D1, CDK4, and pRb) involved in cell cycle progression [59,60]. Western blotting of cells treated with CAPE-MotAb revealed a stronger upregulation of p53, a major tumor suppressor protein (Figure 5B). Cell cycle progression is strictly controlled by cyclins and cyclin-dependent kinases (CDKs). The activated cyclin/CDK complexes (CDK4 and CDK6 associate with D-type cyclins) phosphorylate and inactivate members of the retinoblastoma (Rb) protein family, resulting in progression of the cell cycle [61]. In line with the stronger upregulation of p53 and its effector p21^WAF1^ in CAPE-MotAb treated cells, they showed a higher decrease in the expression of Cyclin D1, CDK4, and pRb as compared to CAPE-PEG treated cells. These results supported that the stronger growth arrest in CAPE-MotAb treated cells is mediated by enhanced activation of p53-p21 and inhibition of pRb signaling pathways.

We next performed Annexin V/7-AAD double staining to evaluate the apoptosis in CAPE-PEG and CAPE-MotAb treated cells. Cell populations in Annexin V-/7- ADD-, Annexin V+/7-ADD-, Annexin V+/7-ADD+, and Annexin V-/7-ADD+ represent healthy, early apoptotic, late apoptotic and debris cells, respectively. Annexin V positive (early and late apoptosis) cells were considered as the apoptotic population. As shown in Figure 5C, apoptosis was significantly enhanced in CAPE-MotAb treated A549 cells as compared to CAPE-PEG treated cells. With the use of MotAb, the apoptotic cell population increased from 29% (CAPE-PEG) to 45% (CAPE-MotAb) (Figure 5C). To determine the molecular mechanism of CAPE-MotAb induced apoptosis, we analyzed the expression of proteins involved in apoptosis. These included polyADP-ribose polymerase-1 (PARP-1), B-cell lymphoma 2 (Bcl-2), Caspase 3, Caspase 9, Cytochrome C, and Bax that are known to be tightly involved in the apoptotic signaling pathway (Figure 5D). Suppression of the anti-apoptotic factors or activation of the pro-apoptotic members of the Bcl-2 family leading to altered mitochondrial membrane permeability resulting in the release of cytochrome c into the cytosol is a well-known mechanism. Binding of Cytochrome C to apoptotic protease activating factor 1 (Apaf-1) triggers the activation of Caspase 9, which then accelerates apoptosis by activating other caspases. Western blot analysis revealed that the downregulation of Bcl2, pro-caspase 3, and pro-caspase 9 was stronger in CAPE-MotAb as compared to CAPE-PEG treated cells (Figure 5D). Whereas the expression level of PARP-1 and Cytochrome C proteins was strongly downregulated, Bax (pro-apoptosis marker) was upregulated to a larger extent in A549 cells exposed to CAPE-MotAb as compared to CAPE-PEG (Figure 5D). Taken together, these results demonstrated that CAPE exerted anticancer activity through induction of cell cycle arrest and apoptosis, and CAPE-MotAb nanoparticles offered higher potency.

### 3.5. CAPE-MotAb Caused Enhanced Anti-Migration and Anti-Invasion Activities

**Figure 6:**
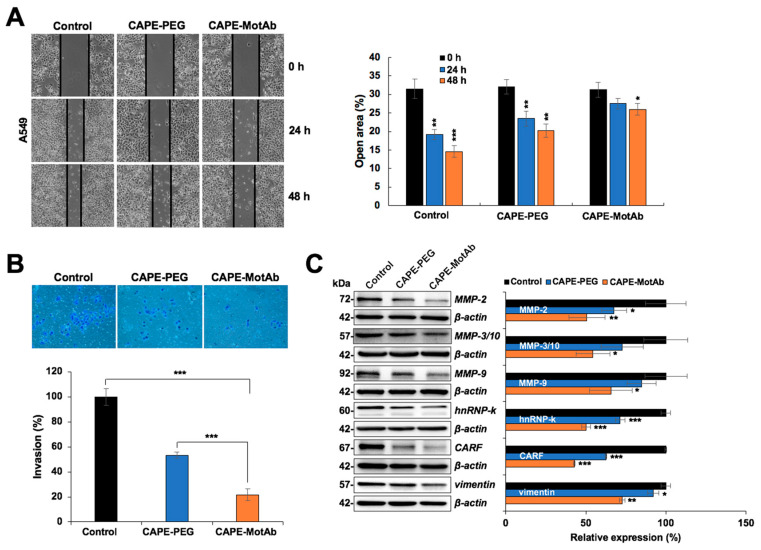
CAPE-MotAb possesses enhanced anti-migration and anti-invasion activity. (**A**) Wound scratch assay showing that CAPE-MotAb treated A549 cells moved slower when compared to the other two groups as indicated in the open area of the wound (CAPE concentration: 5 μg/mL). Quantitation of the open area from three independent experiments (mean ± SD, *n* = 3), * *p* < 0.05, ** *p* < 0.01, *** *p* < 0.001 (Student’s t-test to control). (**B**) Representative images of the invasion assay exhibiting impaired invasion in CAPE-MotAb treated A549 cells. Quantitation of cell invasion results from three independent experiments (mean ± SD, *n* = 3), *** *p* < 0.001 (Student’s t-test). A549 cells were treated with an equivalent dose of CAPE (10 µg/mL) for 24 h. (**C**) Western blot analysis for metastasis-associated proteins (MMP2, MMP3, MMP9, hnRNP-k, CARF, and Vimentin) after 48 h incubation of cells with CAPE-MotAb (10 µg/mL). Quantitation of the results is shown on the right (mean ± SD, *n* = 3), * *p* < 0.05, ** *p* < 0.01, *** *p* < 0.001 (Student’s t-test to control).

CAPE has earlier been shown to inhibit cancer cell migration. We, therefore, investigated the effect of CAPE-MotAb nanoparticles on cancer cell migration and invasive abilities by wound scratch and cell invasion assays, respectively. Microscopic observations of the motility of living cells in the scratch area revealed that cell migration was significantly delayed in CAPE-MotAb treated cells compared to CAPE-PEG treated counterparts (Figure 6A). We observed that the gap area in A549 cells treated with CAPE-MotAb for 24 and 48 h (28% and 26%, respectively) was larger than that of CAPE-PEG (23% and 20%, respectively) from the initial value of 32%, suggesting that CAPE-MotAb caused stronger inhibition of cancer cell migration (Figure 6A). Similarly, in the cell invasion assay, CAPE-MotAb treatment markedly attenuated the invasion ability of A549 cells (Figure 6B). The number of invasive A549 cells was significantly decreased from 53% in CAPE-PEG to 22% in CAPE-MotAb treated cells (Figure 6B). In order to test this further, we next examined the expression level of key metastasis-regulatory proteins and found a significant decrease in MMP-2, MMP-3, MMP-9, CARF, and Vimentin in CAPE-MotAb treated A549 cells (Figure 6C). Furthermore, the level of hnRNP-K (pro-metastasis marker) was also evidently downregulated in cells subjected to CAPE-MotAb treatment (Figure 6C). Of note, CAPE-MotAb treated cells showed a significant decrease in the expression of metastasis-regulatory protein markers as compared to CAPE-PEG treated cells. Taken together, these results demonstrated that CAPE-MotAb nanoparticles possessed enhanced anti-migration and anti-invasion activity as compared to CAPE alone.

### 3.6. CAPE-MotAb Nanoparticles Caused Enhanced Tumor Suppression

**Figure 7:**
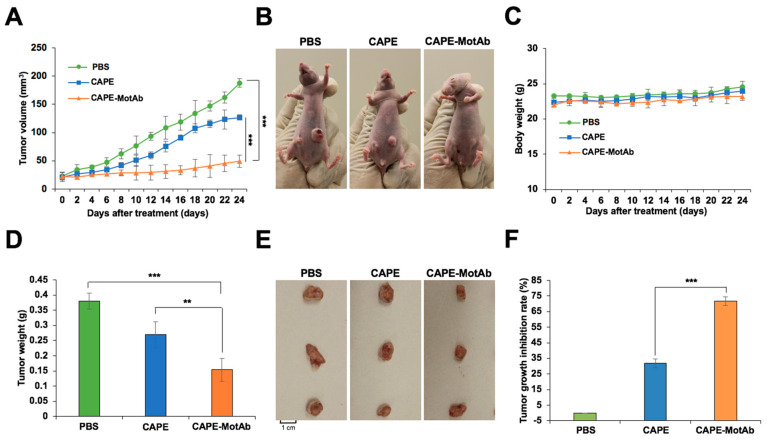
Enhanced selective tumor-suppression activity of CAPE-MotAb in in vivo conditions. (**A**) Relative tumor volumes of A549 cells in the control, CAPE, and CAPE-MotAb treated mice (mean ± SD, *n* = 4), *** *p* < 0.001 (Student’s t-test). The tumor-forming ability in nude mice bearing A549 xenografts treated by CAPE-MotAb was significantly decreased when compared to control and CAPE treated groups. (**B**) Images showing a greater reduction in tumor volume after treatment with CAPE-MotAb compared to the other two groups. (**C**) No significant changes in body weight were observed during the treatment period. The average weights (**D**) and images (**E**) of A549 xenograft tumors dissected at the experiment endpoint (mean ± SD, *n* = 4), ** *p* < 0.01, *** *p* < 0.001 (Student’s t-test). (**F**) Tumor growth inhibition rate following treatment with CAPE and CAPE-MotAb in A549 xenografts (mean ± SD, *n* = 4), *** *p* < 0.001 (Student’s t-test). All the formulations were administered intraperitoneally with an equivalent dose of CAPE (200 mg/Kg/2 days).

We finally investigated the antitumor efficacy of CAPE-MotAb in in vivo conditions by employing nude mice A549 subcutaneous xenograft models. For this purpose, CAPE-MotAb nanoparticles were prepared freshly and subjected to intraperitoneal injection in A549 xenograft nude mice. A549 tumor-bearing nude mice, with an initial average tumor volume of 22 mm^3^, were randomly divided into three groups. The control group was administered with PBS only. The second and third groups were administered with an equivalent dose (200 mg/kg body weight) of either CAPE (known to possess anticancer activity) or CAPE-MotAb nanoparticles. The average tumor volume in PBS-treated mice rapidly increased and reached 188 mm^3^ at the end of the experiment (24 days). Notably, the average tumor volume of CAPE and CAPE-MotAb treated mice were 127 mm^3^ and 49 mm^3^, respectively, at 24 days (Figure 7A). Tumor growth suppression observed in the CAPE-MotAb treated group was significantly higher than the control and CAPE groups. The data strongly supported that the CAPE-MotAb nanoparticles possess enhanced tumor suppressor activity (Figure 7B). No significant change in the bodyweight of mice throughout the treatment period was observed suggesting the lack of toxicity or negative effects of the treatment in any group (Figure 7C). At the endpoint, the mice were sacrificed, and their tumors were excised and weighed. The average tumor weight decreased from 0.38 g in the control to 0.27 g (CAPE) and 0.15 g (CAPE-MotAb), respectively (Figure 7D). A significant reduction in tumor size was observed at the endpoint in the group treated with CAPE-MotAb, when compared to control and CAPE groups (Figure 7E). Following the above assay, tumor tissue lysates were subjected to Western blotting for proteins involved in cell migration/tumor malignancy and metastasis. As shown in Figure S6, we found that the tumors from the group that were treated with CAPE-MotAb possessed significantly lower expression levels of proteins involved in malignant transformation of cells including, MMP-2, MMP-3/10, MMP-9, CARF, and mortalin. Furthermore, as shown in Figure 7F, the tumor growth inhibition rate in response to CAPE-MotAb treatment reached 71.7% in the A549 xenografts, which was much higher than those treated with CAPE (31.8%). These data clearly demonstrated that CAPE-MotAb nanoparticles possessed higher selectivity and cytotoxicity for cancer cells both in in vitro and in vivo. Anticancer activity of CAPE has earlier been assigned to its several mechanisms of action including activation of tumor suppressor and apoptotic activities of p53 protein [11,18,28,30,62,63], modulation of the redox state of cells [64], restoration of gap junctional intercellular communication [65], inhibition of VEGF-induced angiogenesis [66], inhibition of EMT [67], radio- and chemo-sensitization of cancer cells [16,68,69], inhibition of inflammation signaling [70], activation of MAPK-ERK1/2 signaling [71] and inactivation of oncogenic PAK1 signaling [72]. 

## 4. Discussion

CAPE has been identified as an active phenolic compound in New Zealand propolis and assigned a variety of therapeutic activities, including modulation of proteins and pathways molecules involved in immune response and cancer [11,20,21,22,23,24,25,26,27,28]. However, poor water-solubility of phenolic compounds has been documented to limit their efficacy [73]. In addition, bioavailability and hence the therapeutic action of CAPE falls off due to its degradation by cellular esterases [30,31]. Our previous study demonstrated that CAPE can be protected from esterases by its combination with γCD, and hence exhibited stronger anticancer activity [30,31]. It has been widely shown that through the modification of physicochemical properties (size, shape, composition, surface charge, ligands, hydrophilicity, and hydrophobicity), the natural or synthetic molecules and their derivatives can achieve better therapeutic potential [37,38,39]. In order to overcome poor water solubility and low bioavailability that limits its therapeutic efficacy, we currently considered to develop nanoparticles that could potentially serve as nanomedicine [74]. Polyethylene glycol (PEG)-coated polymer has been shown to serve as nanocarriers [75,76,77]. Based on the fact that mortalin is overexpressed on the surface of aggressive and metastatic cancer cells [49,50,51], we recruited DSPE-PEG-NHS and unique anti-mortalin antibody (that possesses cell-internalization characteristics) to target, these nanoparticles selectively to cancer cells. 

Particle size, encapsulation and loading efficiency of CAPE-MotAb nanoparticles was investigated by physical and chemical assays. Encapsulation and loading efficiency of the nanoparticles were both satisfactory with a ratio of 1:5 for CAPE and DSPE-PEG-NHS (Table 1 and Table 2). The successful conjugation of MotAb to DSPE-PEG-NHS and encapsulation of CAPE in MotAb-conjugated polymeric micelles were confirmed by non-reducing SDS-PAGE analysis and UV-Vis-NIR spectrum of CAPE-MotAb (Figure 1). Cell based assays revealed higher cytotoxicity of CAPE-MotAb in cancer cells in which mortalin is enriched on the cell surface (Figure 2 and Figure 3). A549 cells have higher cellular uptake of CAPE-MotAb nanoparticles in a time-dependent manner as observed by fluorescence microscopy-based uptake study in vitro (Figure 4 and Figure S5). CAPE-MotAb nanoparticles cause stronger cell cycle arrest and apoptosis as analyzed by flow cytometry (Figure 5). Enhanced anti-migration and anti-invasion activities are also shown in CAPE-MotAb nanoparticles- treated cancer cells (Figure 6). Furthermore, tumor suppression in animal model provided evidence that CAPE-MotAb nanoparticles possess better anti-tumor activity (Figure 7 and Figure S6). Taken together, all these experimental results support our hypothesis that MotAb-conjugated nanoparticles can specifically deliver CAPE to cancer cells that are enriched in mortalin on the cell surface. Presently, PEGylation has become a mainstay in nanoparticle formulation and PEGylated-nanoparticle based nanoformulations are approved by FDA [76]. Considering the observations made in the present study, we believe that the targeted CAPE delivery approach using MotAb-conjugated nanoparticles holds considerable potential with better outcomes, initiating further studies in a preclinical setup.

## 5. Conclusions

In conclusion, we have developed a simple, water-soluble, and cell internalizing CAPE-MotAb nanoparticles with enhanced targeted delivery and selective cytotoxicity to cancer cells in vitro and in vivo. We, therefore, propose CAPE-MotAb as a suitable nanomedicine and a platform to facilitate the use of caffeic acid phenethyl ester for cancer treatment and hence warrant clinical trials.

## Figures and Tables

**Table 1:** Encapsulation efficiency of CAPE-MotAb prepared with different weight ratios of CAPE powder and DPSE-PEG-NHS polymers.

CAPE/Polymer (*w*/*w*)	Encapsulation Efficiency (%)
1:0.5	27.74 ± 0.58
1:1	38.29 ± 2.24
1:2	40.41 ± 1.06
1:4	71.12 ± 6.94
1: 5	75.88 ± 1.99
1: 10	80.91 ± 7.58
1: 20	84.88 ± 8.66
1: 30	77.21. ± 6.78

**Table 2:** Loading efficiency of CAPE-MotAb prepared with different weight ratios of CAPE powder and DPSE-PEG-NHS polymers.

CAPE/Polymer (*w*/*w*)	Loading Efficiency (%)
1:0.5	18.50 ± 0.39
1:1	19.65 ± 0.96
1:2	13.47 ± 0.35
1:4	14.22 ± 0.14
1: 5	12.65 ± 0.33
1: 10	7.36 ± 0.69
1: 20	4.04 ± 0.41
1: 30	2.49 ± 0.22

## Supplementary Materials

The following are available online at https://www.mdpi.com/2072-6694/12/9/2370/s1, Figure S1: UV-Vis-NIR absorption spectra of CAPE-MotAb nanoparticles at different time points (0 and 8 days), Figure S2: Viability of (A) A549 and (B) MRC5 cells treated with DSPE-PEG-NHS and MotAb (at the concentrations indicated) for 24 h and 48 h, Figure S3: Cytotoxic effect of CAPE, CAPE-PEG, and CAPE-MotAb on (A) A549, (B) HCT116, and (C) HT-29 cells at the concentrations indicated for 24 h and 48 h, respectively, Figure S4: Fluorescence microscopy images of A549 and MRC5 cells treated with DSPE-PEG-NHS and MotAb for 12 h followed by staining with Alexa FluorTM 594-tagged secondary antibody, Figure S5: Fluorescence microscopy images of A549 cells treated with CAPE-PEG and CAPE-MotAb for 24 and 48 h respectively, Figure S6: Western blotting analysis of tumor lysates.
