# Supplementary material for: Novel Caffeic Acid Phenethyl Ester-Mortalin Antibody Nanoparticles Offer Enhanced Selective Cytotoxicity to Cancer Cells
Source: Cancers (Basel). 2020 Aug 21;12(9):2370. doi: 10.3390/cancers12092370 (PMC7564736; doi:10.3390/cancers12092370)
Supplement: Supplementary file 1 [file cancers-12-02370-s001.pdf]

Supplementary Materials I

Novel Caffeic Acid Phenethyl Ester-Mortalin Antibody Nanoparticles Offer Enhanced Selective Cytotoxicity to Cancer Cells

Jia Wang, Priyanshu Bhargava, Yue Yu, Anissa Nofita Sari, Huayue Zhang, Noriyuki Ishii, Kangmin Yan, Zhenya Zhang, Yoshiyuki Ishida, Keiji Terao,Sunil C. Kaul, Eijiro Miyako and Renu Wadhwa

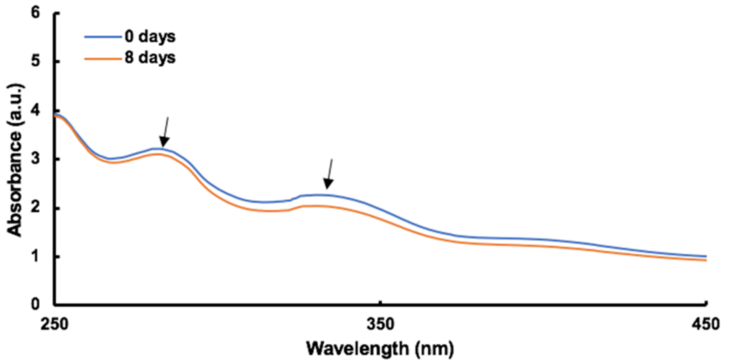

Figure S1. UV-Vis-NIR absorption spectra of CAPE-MotAb nanoparticles at different time points (0 and 8 days). Absorbance of MotAb at 280 nm and CAPE at 335 nm (marked with black arrows) didn't show significant difference from 0 to 8 days.

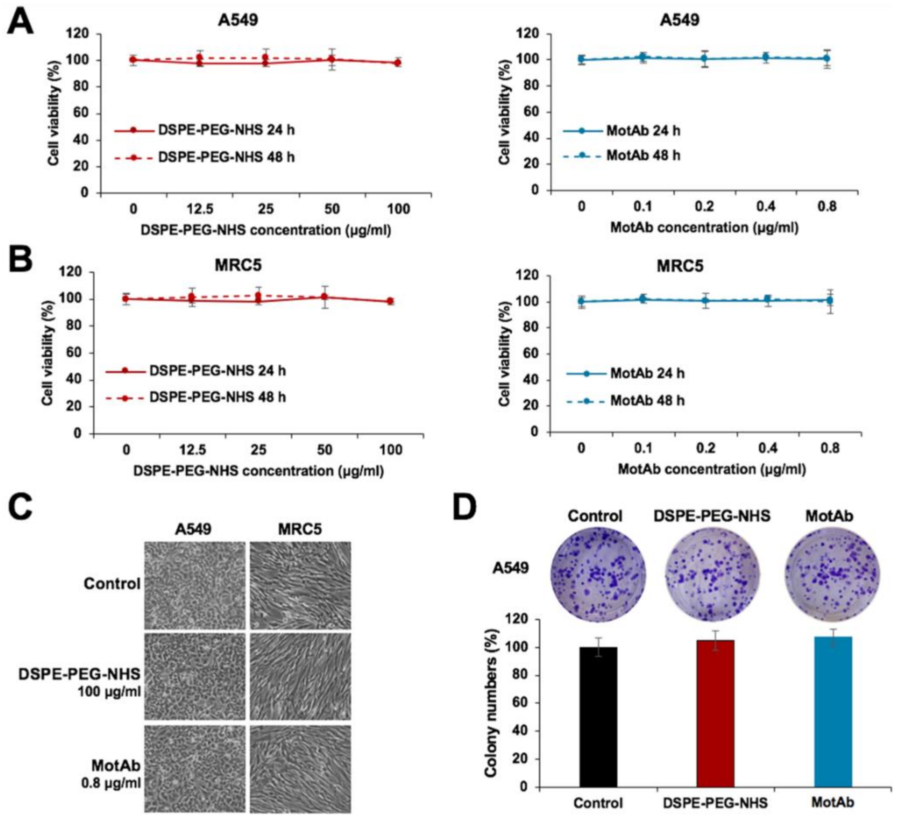

Figure S2. Viability of **(A)** A549 and **(B)** MRC5 cells treated with DSPE-PEG-NHS and MotAb (at the concentrations indicated) for 24 h and 48 h. No cytotoxic effect of DSPE-PEG-NHS and MotAb was obtained on A549 and MRC5 cells (mean ± SD, *n* = 3). **(C)** Phase contrast images show no toxicity

when A549 and MRC5 cells exposed to DSPE-PEG-NHS and MotAb. **(D)** No reduction in colony forming ability was observed in treated cells after 12 h incubation. DSPE-PEG-NHS concentration: 100 μg/mL, MotAb concentration: 0.8 μg/ml. Quantitation from three independent experiments (mean ± SD, *n* = 3).

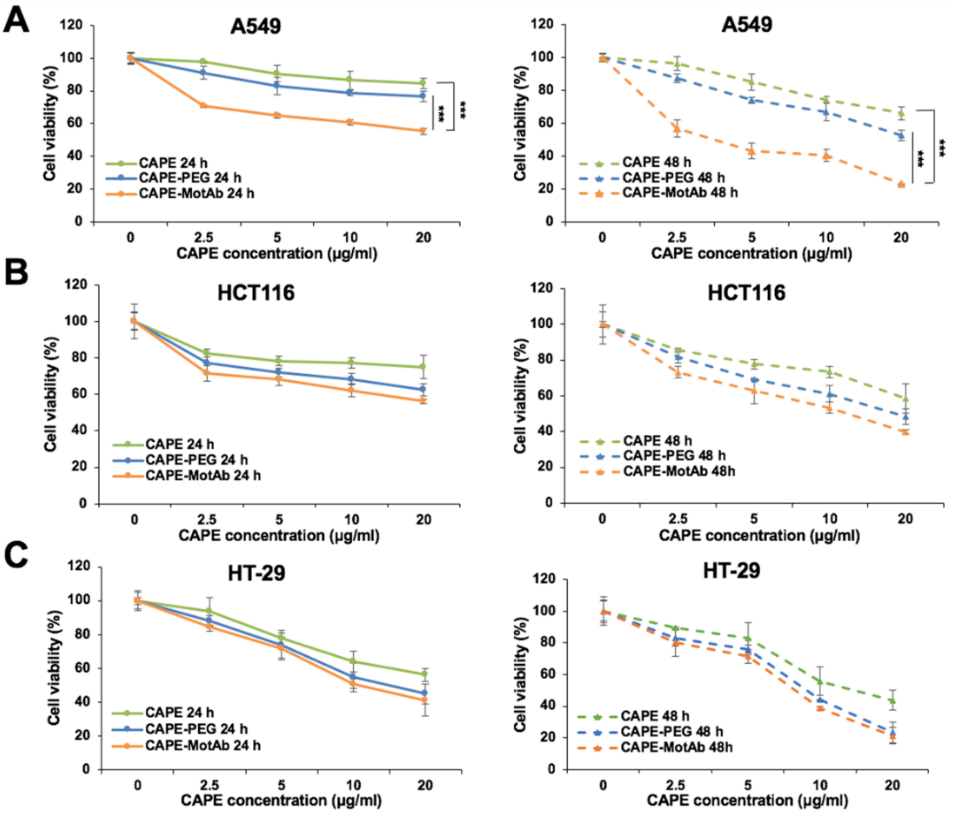

Figure S3. Cytotoxic effect of CAPE, CAPE-PEG and CAPE-MotAb on (**A**) A549, (**B**) HCT116 and (**C**) HT-29 cells at the concentrations indicated for 24 h and 48 h, respectively. These three cell lines treated with CAPE, CAPE-PEG and CAPE-MotAb showed dose- and time-dependent decrease in cell viability. CAPE-MotAb treated A549 cells showed enhanced cytotoxicity as compared to CAPE and CAPE-PEG treatments; HCT116 and HT-29 cells did not show much difference. Quantitation from three independent experiments (mean ± SD, *n* = 3)**,** *** $p < 0.001$ (Student's t-test).

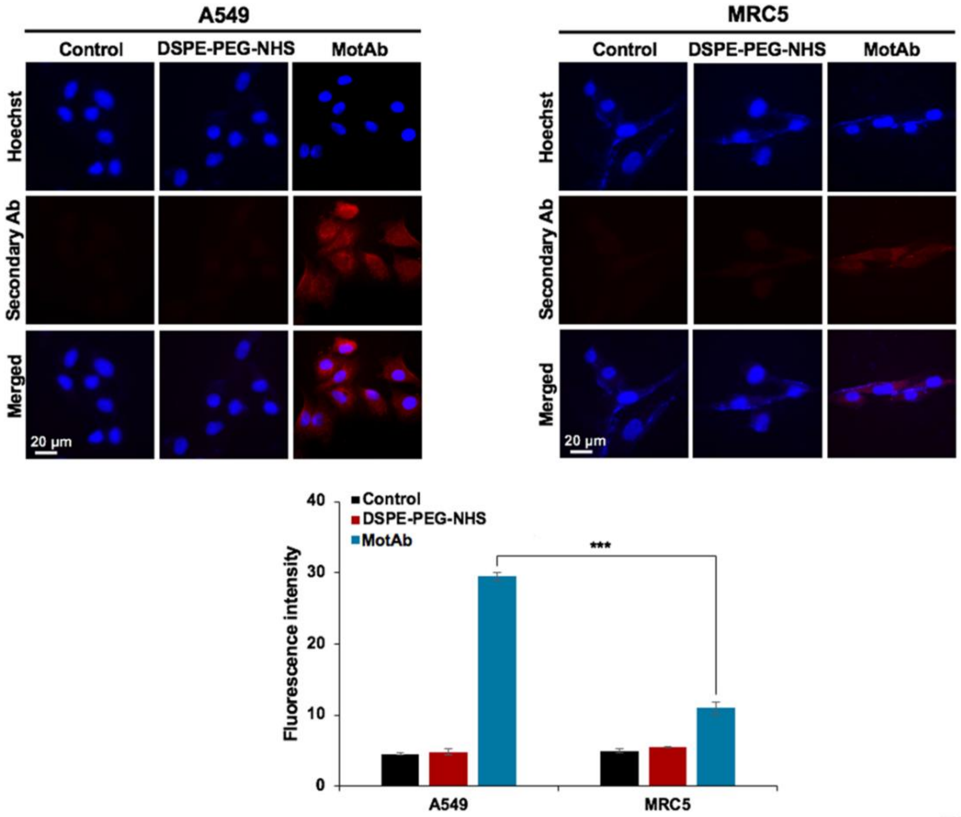

Figure S4. Fluorescence microscopy images of A549 and MRC5 cells treated with DSPE-PEG-NHS and MotAb for 12 h followed by staining with Alexa Fluor™ 594-tagged secondary antibody. The nuclei were stained with Hoechst. DSPE-PEG-NHS concentration: 100 μg/mL, MotAb concentration: 0.8 μg/mL. Quantitation of mortalin expression from fluorescence images (mean ± SD, $n = 3$), *** $p < 0.001$ (Student's t-test).

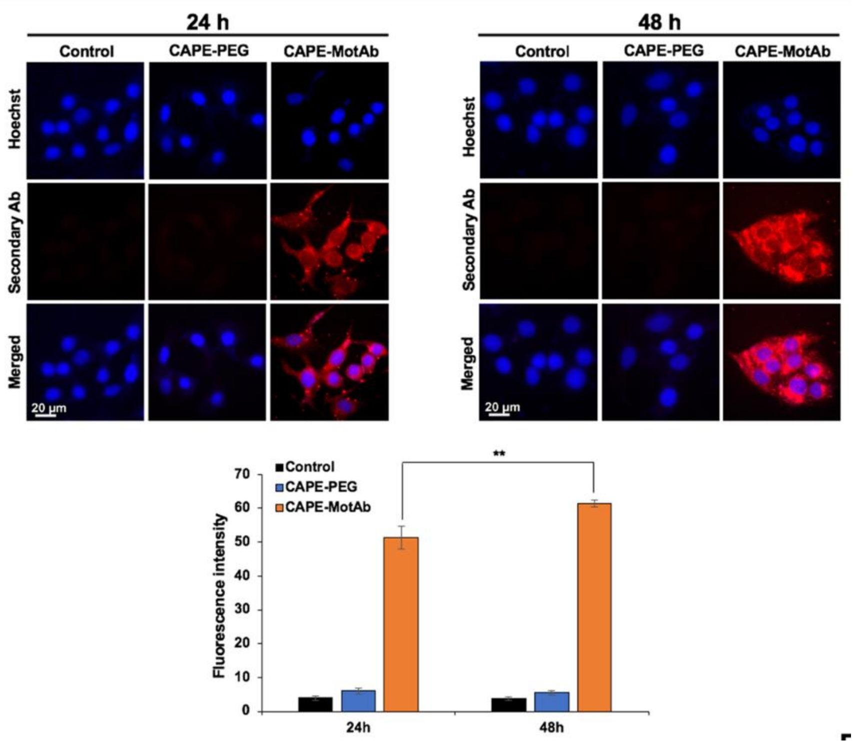

Figure S5. Fluorescence microscopy images of A549 cells treated with CAPE-PEG and CAPE-MotAb for 24 and 48 h respectively, followed by staining with Alexa Fluor™ 594-tagged secondary antibody. The nuclei were stained with Hoechst. Cellular uptake of CAPE-MotAb by A549 cells was in a time-

dependent manner. CAPE concentration: 20 μg/mL. Quantitation of mortalin expression from fluorescence images (mean ± SD, *n* = 3), ** p < 0.01 (Student's t-test).

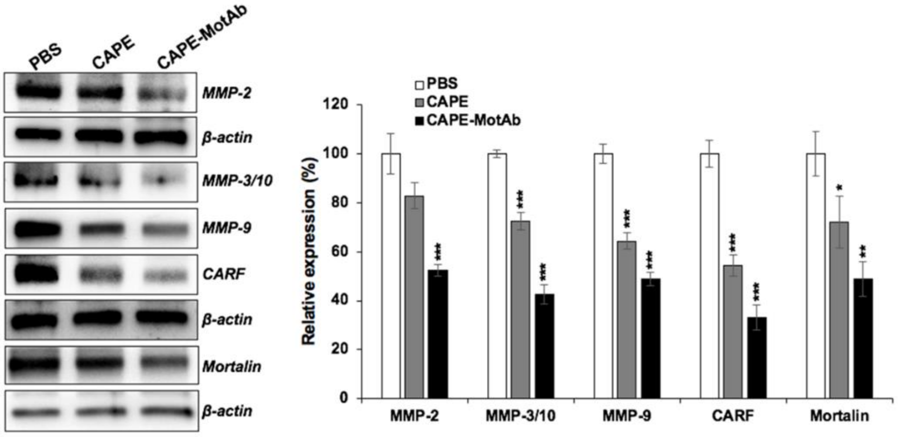

Figure S6. Western blotting analysis of tumor lysates. CAPE-MotAb treated group showed significant downregulation of MMP-2, MMP-3/10, MMP-9, CARF and mortalin levels. Quantitation from three independent experiments (mean ± SD, $n = 3$), * $p < 0.05$, ** $p < 0.01$, *** $p < 0.001$ (Student's t-test to control).
